# A11 FERMENTABLE DIETARY FIBER INTAKE IS ASSOCIATED WITH A DECREASED RISK OF DEVELOPING CROHN’S DISEASE IN HEALTHY FIRST-DEGREE RELATIVES

**DOI:** 10.1093/jcag/gwae059.011

**Published:** 2025-02-10

**Authors:** C McShane, M Xue, J Kim, H Leibovitzh, J Shao, Q LI, R Khorasaniha, K Madsen, A Griffiths, P Moayyedi, S Lee, H Armstrong, K Croitoru, W Turpin

**Affiliations:** Sinai Health, Toronto, ON, Canada; Sinai Health, Toronto, ON, Canada; Sinai Health, Toronto, ON, Canada; Sinai Health, Toronto, ON, Canada; Sinai Health, Toronto, ON, Canada; Sinai Health, Toronto, ON, Canada; University of Manitoba, Winnipeg, MB, Canada; University of Alberta, Edmonton, AB, Canada; The Hospital for Sick Children, Toronto, ON, Canada; McMaster University, Hamilton, ON, Canada; Sinai Health, Toronto, ON, Canada; University of Alberta, Edmonton, AB, Canada; Sinai Health, Toronto, ON, Canada; Sinai Health, Toronto, ON, Canada

## Abstract

**Background:**

The cause of Crohn’s Disease (CD) remains unclear; however evidence suggests that diet plays a key role. With a rising incidence and no definitive cure, identification of modifiable risk factors is critical in preventing CD development.

**Aims:**

To study the association between fermentable dietary fiber intake and the future development of CD in an at risk population.

**Methods:**

Participants were recruited in the CCC-GEM study, a prospective cohort study of healthy first-degree relatives of patients with CD. At enrolment, a validated food frequency questionnaire was administered. Pectin, β-glucan, inulin, fructooligosaccharides (FOS), and arabinoxylan intake were quantified, and values energy-adjusted. Survival analysis was used to test the association between intake of fiber subtypes and risk of CD development. Generalized estimating equation was used to test the association between fiber intake and baseline impaired intestinal permeability (fractional urinary excretion of lactulose to mannitol ratio (LMR)>0.025); gut sub-clinical inflammation (fecal calprotectin (FCP)>100 μg/g); and fecal microbiome composition via 16s rRNA sequencing.

**Results:**

Of the 2,659 participants 76 developed CD with a median follow-up of 8.9 years (IQR=5.7-12.3). Median age at recruitment was 17 years (IQR=12-25). 47% were male. Higher intake of inulin (HR = 0.78; 95% CI=0.61–0.99; p=0.04) and β-glucan (HR = 0.78; 95% CI=0.61–0.99; p=0.04) were associated with a reduced risk of CD development. No fiber subtype was associated with a higher risk of CD development. Lower intake of pectin, inulin, FOS and β-glucan was associated with impaired intestinal permeability. Lower inulin intake was associated with high FCP levels. Higher pectin intake was associated with increased alpha diversity. Higher inulin, FOS and pectin intake was associated with a shift in microbial taxa previously shown to be protective against CD onset in the GEM cohort; a reduction in *Ruminococcus torques* and an increase in *NK4A214 group* (phylum *Firmicutes*) presence (3.25×10^−10^<q<0.04).

**Conclusions:**

This study demonstrates that fermentable fiber subtypes are associated with a reduced risk of CD development as well as with pleiotropic changes in CD risk biomarkers including microbiome, intestinal permeability, and subclinical inflammation. This suggests that the potential benefit of fermentable fibers on CD risk may involve changes in barrier function, and microbiome composition and function. Targeted interventions based on these findings could prove effective in preventing the onset of CD.

Submitted on behalf of the Crohn’s and Colitis Canada GEM Project Research Consortium, www.gemproject.ca.

**Funding Agencies:**

